# The Validation of Clinical Examination and MRI as a Diagnostic Tool for Cruciate Ligaments and Meniscus Injuries of the Knee Against Diagnostic Arthroscopy

**DOI:** 10.7759/cureus.15727

**Published:** 2021-06-17

**Authors:** Kumar Shantanu, Shailendra Singh, Shubham Srivastava, Atul K Saroj

**Affiliations:** 1 Department of Orthopedic Surgery, King George's Medical University, Lucknow, IND

**Keywords:** anterior cruciate ligament (acl) injuries, posterior cruciate ligament (pcl) injuries, meniscus injuries, magnetic resonance imaging, clinical examination

## Abstract

Background: This study was conducted to compare the accuracy of MRI findings and clinical examination of ligamentous and meniscal injuries of the knee, taking arthroscopy as a standard diagnostic tool in knee injuries.

Methods: All patients with knee injuries attending the outpatient department or emergency of our hospital underwent clinical examination. Out of them, 60 patients with knee injuries were subjected to clinical examination, MRI, and then arthroscopy. The findings of these diagnostic tools in respect to the anterior cruciate ligament (ACL), posterior cruciate ligament (PCL), and meniscus injuries were validated, compared, and analyzed using various statistical tools. The accuracy, sensitivity, negative predictive value (NPV), positive predictive value (PPV), and specificity were calculated and an agreement between various tests was established using kappa statistics.

Results: The accuracy of clinical examination in our study was 88% for ACL tears, 85% for meniscal tears, and 100% for PCL tears. The kappa measure of agreement between arthroscopy and clinical finding and MRI for ACL was 0.610 and 0.698, respectively, which was statistically significant. MRI (98.1) was found to be a more sensitive test for detecting ACL injury than clinical examination (90.4%) resulting in higher diagnostic accuracy (98.3%), while diagnostic accuracy of clinical examination and MRI was found to be 100% for PCL injuries. Hence, MRI is an excellent screening tool for ligamentous and meniscal injuries of the knee joint. We can avoid diagnostic arthroscopy in patients with knee injuries having equivocal clinical and MRI examinations and can proceed for therapeutic arthroscopy to deal with such injuries.

Conclusions: For the assessment of ligamentous and meniscal injuries, MRI is an accurate and noninvasive modality. It can be used as a first-line investigation but arthroscopy remains the gold standard.

## Introduction

In today's world of technology and advancement, we often come across ill hazards popping out of it. Road traffic accidents are tremendously increasing in number with increasing incidence of various injuries like ligamentous and meniscal injuries within the knee. Many times, we find patient coming with complaints of knee injury from all age groups, from a pedestrian crossing street to elderly falling on the ground, almost all athletes' experiences knee injury during their lifetimes.

The knee joint has a complex structure due to which it is more susceptible to different types of injuries like fracture, dislocation, and tear in the ligaments, tendons, and cartilage. Because of its physical nature, its vulnerability to external forces, and the functional demands imposed on it, the knee is one of the most commonly injured joints [1].

In older times we had limited resources to diagnose and manage cases associated with a knee injury and it was mainly done based on the clinical examination and x-rays. Clinical examinations are used to diagnose knee injuries, with some of them have become standard clinical tests for particular injuries a long time ago [2].

With the advent of radiological advancements, like MRI and CT scans we can look more clearly inside the joint. Above all, MRI provides the advantage of being a fast, non-invasive, diagnostic tool to look for ligament and menisci injuries. Thus, it minimizes the agony and morbidity faced by the patients, along with the ease in the management with better planning and optimal intervention in time. MRI provides a better understanding to identify the ligament, menisci, synovial injury. MRI diagnosed almost all the ligamentous and meniscus injuries with a great level of confidence [3].

Arthroscopic examination of the knee is a more valuable method than diagnosis by MRI and clinical tests for detecting meniscal-cruciate injuries of the knee [4-6]. Arthroscopy has now become the gold standard in diagnosing knee pathologies [5].

The purpose of our study was to validate the findings of clinical examination and MRI as a diagnostic tool for intraarticular knee injuries; anterior cruciate ligament (ACL), posterior cruciate ligament (PCL), and meniscal injuries of the knee against findings of diagnostic arthroscopy.

## Materials and methods

This prospective cohort study was conducted on 60 individuals in the Department of Orthopedics, King George’s Medical University, Lucknow, Uttar Pradesh, India, who had fulfilled the inclusion criteria and had given consent for the study. The study period was one year from February 2019 to February 2020. The study was approved by the institutional ethics committee. 

The study population was having a history of knee injury with suspected anterior cruciate ligament, posterior cruciate ligament, and meniscus injury admitted to the Department of Orthopedics, King George's Medical University. The patients within the age group of 18-45 years having a history of knee injury and suspected traumatic anterior cruciate ligament, posterior cruciate ligament, and menisci injury within eight weeks were included.

The patients with conditions that preclude MRI-like patients having intracerebral aneurysmal clips, cardiac pacemaker, stainless steel implants in bone, patients having symptoms suggestive of tumor of the knee joint structures, patients who underwent arthroscopic examination directly without undergoing MRI scanning, and those who were unfit for anesthesia were excluded from the study.

 All patients underwent clinical, MRI, and arthroscopic examinations. The findings of clinical examination and MRI were compared with diagnostic arthroscopy. In all patients, radiological investigations were done after three weeks of injury. MRI used was of 1.5 Tesla scanner and T1- and T2-weighted and proton dense slices on sagittal and coronal planes were obtained. Their MRI films were read by our radiologist to report; the radiologist was unaware of the finding of the clinical examination. The cruciate ligaments were classified as partial disruption or complete ligament injury. MRI grading system for meniscal injuries was used in this study (Table 1).

**Table 1:** MRI grading system for meniscal injuries

Grade	Signal
Normal	No abnormal signal intensity
1	Small focal area of increased signal intensity, with no extension to the articular surface
2a	Linear abnormal signal intensity with no extension to the articular surface
2b	Abnormal signal intensity reaches the articular surface, but on a single image
2c	Globular wedge-shaped abnormal signal intensity with no extension to the articular surface
3	Abnormal high signal intensity extends to at least one articular surface (superior or inferior). This type is referred to as a definite meniscal tear

All knees were clinically examined for cruciate ligament and menisci injuries. For cruciate ligament examination, anterior drawer test, posterior drawer test, Lachman test, pivot shift test, and posterior sag sign, and for meniscal injury, Mc Murray's tests were performed. All clinical tests were performed by a single investigator to eliminate interpersonal bias.

Arthroscopy was performed by a single operating surgeon having 15 years of experience in knee arthroscopy and who was unaware of clinical and MRI findings. The findings of arthroscopy were considered as true diagnoses.

To classify the location of meniscal tear arthroscopically, each meniscus was divided into three equal segments: (1) the anterior one-third or anterior horn; (2) the middle one-third or body; (3) posterior one-third or posterior horn.

The results were recorded on Microsoft Excel. The number and percentage for qualitative data and mean + SD for quantitative data. In order to assess the reliability and validity of screening tests, sensitivity, specificity, positive predictive value (PPV), negative predictive value (NPV), and diagnostic accuracy were calculated. The agreement between various tests was evaluated by kappa statistics. The data were analyzed using Microsoft Excel and IBM-SPSS software version 20 (Armonk, NY: IBM Corp.). The p-value less than 0.05 was taken as significant.

## Results

In a total of 60 cases, those who had fulfilled inclusion criteria for traumatic cruciate ligaments and meniscal injuries were clinically examined and reviewed with MRI followed by diagnostic arthroscopy and repair/reconstruction. The data were analyzed to calculate true positive, true negative, false positive, and false negatives. Using this, specificity and sensitivity, positive and negative predictive values were calculated with arthroscopic examination as the gold standard for comparison.

Among the enrolled patients, the majority were males, 55 (91.7%), and the rest were females, five (8.3%). The maximum number of patients, 29 (48.3%), were aged between 26 years and 35 years. The mean age of the patients was 29.17±7.98 years. Among the enrolled patients, a maximum of 32 (53.3%) was the victim of road traffic accidents followed by a sports injury and slip-on ground each with 11 (18.3%) cases while 6 (10.0%) cases were of the fall from height.

Clinically ACL injury was found to be positive in 49 (81.7%) cases, MRI had shown torn ACL in 51 (85.0%) cases and arthroscopically it was detected in 52 (86.7%) cases. MRI and arthroscopic diagnosis for ACL matched for positive in 85.0% cases and matched for negative in 13.3% cases while for the clinical examination it was 78.3% and 10%, respectively (Table 2).

**Table 2:** The clinical and MRI finding and arthroscopic findings in ACL tear ACL: anterior cruciate ligament

ACL	Arthroscopically negative	Arthroscopically positive
MRI negative	8 (13.3%)	1 (1.67%)
MRI positive	0 (0.00%)	51 (85.0%)
Clinically negative	6 (10.0%)	5 (8.3%)
Clinically positive	2 (3.3%)	47 (78.3%)

The kappa measure of agreement between arthroscopy and clinical finding and MRI for ACL was 0.610 and 0.698, respectively, which was statistically significant. MRI (98.1) was found to be a more sensitive test for detecting ACL injury than clinical examination (90.4) resulting in higher diagnostic accuracy (98.3) (Table 3).

**Table 3:** The validity of clinical finding and MRI for detecting ACL tear NPV: negative predictive value; PPV: positive predictive value; ACL: anterior cruciate ligament

Parameter	Value for clinical finding	Value for MRI
Kappa value	0.610	0.698
p-Value	<0.001	<0.001
Sensitivity	90.4	98.1
Specificity	75.0	100.0
PPV	95.9	100.0
NPV	54.5	88.9
Diagnostic accuracy	88.3	98.3

Both clinical and MRI findings found PCL injury to be positive in eight (13.3%) cases and it corresponds to the arthroscopic findings (Table 4).

**Table 4:** The clinical and MRI finding and arthroscopic findings in PCL tear PCL: posterior cruciate ligament

PCL	Arthroscopically negative	Arthroscopically positive
Clinically negative	52 (86.7%)	0 (0.0%)
Clinically positive	0 (0.0%)	8 (13.3%)
MRI negative	52 (80.0%)	0 (0.0%)
MRI positive	0 (0.0%)	8 (12.3%)

The kappa measure of agreement between arthroscopy and clinical finding for PCL was perfect, 1.00. The sensitivity, specificity, PPV, NPV, and diagnostic accuracy of clinical diagnosis were all 100% (Table 5).

**Table 5:** The validity of clinical and MRI findings for detecting PCL tear PCL: posterior cruciate ligament

Parameter	Value for clinical test	Value for MRI
Kappa value	1.000	1.000
p-Value	0.000	0.000
Sensitivity	100.0	100.0
Specificity	100.0	100.0
PPV	100.0	100.0
NPV	100.0	100.0
DA	100.0	100.0

The medial meniscus injury was found clinically in 10 (16.7%) cases while on MRI, it was found in 23 (38.3%) cases and arthroscopically, it was detected in 19 (31.7%) cases (Table 6).

**Table 6:** The medial meniscus injury result summary

Test	Medial meniscus	No. (N=60)	%
Clinically	Negative	50	83.3
	Positive	10	16.7
MRI	Negative	37	61.7
	Positive	23	38.3
Arthroscopically	Negative	41	68.3
	Positive	19	31.7

The clinical and arthroscopy diagnosis for medial meniscus matched positive for nine (15.0%) cases while it was matched positive for 17 (28.33%) with MRI findings. Ten (16.67%) cases were clinically negative but found positive in arthroscopy while one case was clinically positive and negative in arthroscopy (Table 7). High numbers of false-positive meniscal injury were detected with MRI which can be attributed to the fact that signal of T2-weighted images detects mostly superficial surfaces of menisci.

**Table 7:** The clinical, MRI, and arthroscopic findings in medial meniscus injury

Medial meniscus	Arthroscopically negative	Arthroscopically positive
Clinically negative	40 (66.67%)	10 (16.67%)
Clinically positive	1 (1.67%)	9 (15.0%)
MRI negative	35 (58.33%)	2 (3.33%)
MRI positive	6 (10.00%)	17 (28.33%)

The kappa measure of agreement between clinical findings and MRI with arthroscopy for medial meniscus was 0.621 and 0.622, respectively, and was significant (p<0.001) (Table 8). Our results show clinical tests were a more specific tool while MRI was the more sensitive tool for detection of medial meniscus injury. 

**Table 8:** The validity of clinical and MRI findings for detecting medial meniscus NPV: negative predictive value; PPV: positive predictive value; DA: delayed assessment

Parameter	Value for clinical tests	Value for MRI
Kappa value	0.621	0.622
p-Value	<0.001	<0.001
Sensitivity	47.4	89.5
Specificity	97.6	85.4
PPV	90.0	73.9
NPV	80.0	94.6
DA	81.7	86.7

The lateral meniscus was found to be positive clinically in five (8.3%) cases, MRI found in 10 (16.7%) cases, while arthroscopically, it was detected in eight (13.3%) cases (Table 9).

**Table 9:** The lateral meniscus injury result summary

Test	Lateral meniscus	No. (N=60)	%
Clinically	Negative	55	91.7
	Positive	5	8.3
MRI	Negative	50	83.3
	Positive	10	16.7
Arthroscopically	Negative	52	86.7
	Positive	8	13.3

The clinical and arthroscopy diagnosis for lateral meniscus matched positive for four (6.67%) cases and matched negative for 51 (85.0%) cases. Further, four (6.67%) cases were positive in arthroscopic findings but clinically negative while one case was negative in arthroscopy but clinically positive (Table 10).

**Table 10:** The clinical, MRI, and arthroscopic findings in lateral meniscus injury

Lateral meniscus	Arthroscopically negative	Arthroscopically positive
Clinically negative	51 (85.00%)	4 (6.67%)
Clinically positive	1 (1.67%)	4 (6.67%)
MRI negative	49 (81.67%)	1 (1.67%)
MRI positive	3 (5.00%)	7 (11.67%)

The kappa measure of agreement between clinical findings and MRI with arthroscopy for lateral meniscus was 0.780 and 0.785, respectively, which was statistically significant (p<0.001). The clinical tests have proven to be more specific for the diagnosis of lateral meniscus injury while MRI was the most sensitive test to detect lateral meniscus injury similarly as we have found in cases of medial meniscus injury (Table 11).

**Table 11:** The validity of clinical and MRI findings for detecting lateral meniscus NPV: negative predictive value; PPV: positive predictive value; DA: delayed assessment

Parameter	Value for clinical test	Value for MRI
Kappa value	0.780	0.785
p-Value	<0.001	<0.001
Sensitivity	50.0	87.5
Specificity	98.1	94.2
PPV	80.0	70.0
NPV	92.7	98.0
DA	91.7	93.3

## Discussion

The proper history taking and clinical examination are needed for effective clinical decision-making, which further determines whether the patient needs more investigations or therapeutic intervention, which is beneficial for patients ergonomically as well as the time of his morbidity.

In the past, the clinical examination had a significant role in the diagnosis of knee joint injuries, but with the invention of MRI and because of very few side effects, non-invasiveness and progressive growth in technology made it an important tool of diagnosis.

Among 60 patients, 55 were males and five were females of 18-45 years age group having clinically suspected traumatic ligamentous and meniscal injuries, we found that it was more common in males of 26-35 years age group. A study by Avcu et al. found that knee injuries were common in the male population due to their active participation in outdoor works and sports activities [7]. In our study right knee is more involved than the left knee due to the dominating side in most sportspersons.

We found that sensitivity, specificity, PPV, NPV, and accuracy of clinical examinations performed concerning arthroscopy for an ACL tear is 90.4%, 75.0%, 95.9%, 54.5%, and 83.3%, respectively. A study performed by Panigrahi et al. found that the sensitivity, specificity, PPV, NPV, and accuracy of clinical tests were 94.7%, 71.4%, 90.0%, 83.3%, and 88.5%, respectively [2]. The negative predictive value in our study is less due to the five false-negative cases which were not found during a clinical examination. Out of five patients, two were of chronic anterior cruciate ligament tear; when viewed arthroscopically, we found the stump fibrosis of torn anterior cruciate ligament fibers and its attachment with posterior cruciate ligament which result in negative clinical tests; three patients were of partial anterior cruciate ligament tear on arthroscopy which was also negative on clinical examination. Two patients were positive on clinical examination but arthroscopically normal ACL was found in them. These patients were of 44 years and 45 years of age having a history of trauma presented with knee pain, but on arthroscopy, all ligaments and menisci were normal with some early arthritic changes. A study done by Madhusudhan et al. found that knee with degenerative changes can give false-positive results on clinical examination [8].

We analyzed the MRI films of these anterior cruciate ligament injury suspected patients and found that sensitivity, specificity, PPV, NPV, and DA of MRI with respect to arthroscopy 98.1%, 100.0%, 100.0%, 88.9%, and 98.3%, respectively. Previous studies found the sensitivity of 66-100% [3,8-11], specificity of 67-98% [3,8,12], PPV of 75-93% [3,8], NPV of 79-100% [3,8,10], and accuracy of 78-98% [3,8,13]. In our study, we found the specificity and PPV of 100.0% which is due to the absence of false-positive cases on MRI. Kulkarni et al. in their study found the sensitivity, specificity, PPV, NPV, and accuracy of MRI 90.90%, 78.26%, 93.33%, 72.0%, and 88.0%, respectively [9]. Panigrahi et al. found in their study the sensitivity, specificity, NPV, and accuracy of MRI 94.7%, 78.6%, 92.3%, 84.6%, and 90.4%, respectively [2]. In our study the sensitivity, specificity, and PPV of MRI were found more, this can be due to less number of our sample size and more young patients included in our study.

For the posterior cruciate ligament injury, we did the clinical examination on the same patients and found that only eight out of 60 patients were of suspected PCL injury clinically. All of the eight patients when undergone MRI, every patient had been detected with PCL injury which was further confirmed on arthroscopy. In our study, we found the sensitivity, specificity, PPV, NPV, and diagnostic accuracy of diagnosing the PCL injury on clinical examination and MRI was 100% for each. A study performed by Gimhavanekar et al. showed the sensitivity, specificity, PPV, and NPV for PCL tear were 100% each on MRI [3]. Panigrahi et al. in their study also found 100% sensitivity, specificity, PPV, NPV, and accuracy each on clinical examination, but on MRI, the sensitivity, specificity, PPV, NPV, and accuracy were less than 100%.

For the medial meniscus injury, we performed the McMurray test and compare it with arthroscopy and found the sensitivity, specificity, PPV, NPV, and diagnostic accuracy of 47.4%, 97.6%, 90.0%, 80%, and 81.7, respectively. Panigrahi et al. found sensitivity, specificity, PPV, NPV, and diagnostic accuracy of clinical tests for medial meniscus were 76.5%, 68.6%, 54.2% 85.7%, and 71.2%, respectively [2]. Chandru et al. studied the clinical and arthroscopic correlation of medial meniscal injuries of the knee and found the sensitivity and specificity of 83.33% and 77.78%, respectively [10]. Sharma et al. conducted a study on 41 patients to correlate the clinical and MRI finding with arthroscopy and found that the sensitivity, specificity, and diagnostic accuracy of clinical examination for medial meniscus injury were 96.1%, 33.3%, and 73.1%, respectively [11]. In comparison to these studies, the sensitivity of clinical examination for medial meniscus is less in our study, it was due to the 10 patients which came false negative on clinical examination found positive on diagnostic arthroscopy.

After an MRI scan of the same patient, we found the sensitivity, specificity, PPV, NPV, and diagnostic accuracy of 89.5%, 85.4%, 73.9%, 94.6%, and 86.7%, respectively. While in the study by Panigrahi et al., their values were 88.2%, 62.8%, 53.6%, 91.7%, and 71.2%, respectively [2]. Chandru et al. found the sensitivity and specificity of MRI with respect to arthroscopy are 91.67% and 55.56%, respectively [10]. Sharma et al. found the sensitivity, specificity, and diagnostic accuracy of MRI for medial meniscus injury were 92.5%, 100%, and 95.1%, respectively [11]. Previous literature showed the sensitivity of 50-100% [1,2,8,12,13], specificity of 44-100% [2,10,11,14,15], PPV of 71-90% [3,16], NPV of 86-100% [2,14], and diagnostic accuracy of 65-98% of MRI for medial meniscus injury [2,3,11,14,16]. In our study, most of the patients who were false positive on MRI were of grade I medial meniscus tear. Our findings were according to the literature and showed MRI as a good diagnostic tool for medial meniscus injuries in comparison to clinical examination.

On co-relating the findings of clinical examination for lateral meniscus with respect to arthroscopy, we found the sensitivity, specificity, PPV, NPV, and diagnostic accuracy of clinical examination 50%, 98.07%, 80%, 92.72%, and 91.66%, respectively. Panigrahi et al. found the sensitivity, specificity, PPV, NPV, and diagnostic accuracy were 88.2%, 62.8%, 53.6%, 91.7%, and 71.2%, respectively, for clinical examinations [2]. Chandru et al. found sensitivity and specificity of 75% and 77.27%, respectively [10]. Sharma et al. found the sensitivity, specificity, and accuracy of 38.4%, 96.4%, and 78.1%, respectively, for lateral meniscus on clinical examination [11]. In our study on MRI, the sensitivity, specificity, PPV, NPV, and accuracy of the lateral meniscus with respect to arthroscopy were found as 87.5%, 94.2%, 70.0%, 98.0%, and 93.33%, respectively. Paniraghiet al. found these values as 46.7%,89.2%, 63.6%, 80.5%, and 76.9%, respectively [2]. Chandru et al. show the sensitivity and specificity of MRI for the lateral meniscus were 62.5% and 72.73%, respectively [10]. Sharma et al. found the sensitivity, specificity, and accuracy were 86.6%,96.4%, and 92.6%, respectively, on MRI for the lateral meniscus injury [11]. Previous studies showed these values ranges as for sensitivity 41-100% [2,3,11,14,15], specificity 72-100% [2,10,11,14,15], PPV 34-100% [2,14], NPV 90-100% [2,14], and accuracy 68-100% [2,3,11,14,16]. Our results of MRI for diagnosing the lateral meniscus injuries were as per the findings of the literature.

## Conclusions

The accuracy of MRI in the diagnosis of ACL, PCL, and meniscus injuries is exceedingly good. Besides being a noninvasive screening modality, it provides detailed insight and is an essential tool in decision-making before planning for any therapeutic intervention. For the better implications of these results, further studies are warranted with a focus on including using larger sample quantities and multi-centric study.
